# Detrimental Contribution of the Toll-Like Receptor (TLR)3 to Influenza A Virus–Induced Acute Pneumonia

**DOI:** 10.1371/journal.ppat.0020053

**Published:** 2006-06-09

**Authors:** Ronan Le Goffic, Viviane Balloy, Micheline Lagranderie, Lena Alexopoulou, Nicolas Escriou, Richard Flavell, Michel Chignard, Mustapha Si-Tahar

**Affiliations:** 1 Institut Pasteur, Unité de Défense Innée et Inflammation, Paris, France; 2 INSERM, E336, Paris, France; 3 Institut Pasteur, Département de Médecine Moléculaire, Paris, France; 4 Université de la Méditerranée, Faculté des Sciences de Luminy, Centre d'Immunologie de Marseille-Luminy (CIML), Marseille, France; 5 INSERM U631, Marseille, France; 6 CNRS, UMR6102, Marseille, France; 7 Institut Pasteur, Unité de Génétique Moléculaire des Virus Respiratoires, CNRS URA 1966, Paris, France; 8 Section of Immunobiology, Yale University School of Medicine, New Haven, Connecticut, United States of America; 9 Howard Hughes Medical Institute, New Haven, Connecticut, United States of America; University of Texas, United States of America

## Abstract

Influenza A virus (IAV) is the etiological agent of a highly contagious acute respiratory disease that causes epidemics and considerable mortality annually. Recently, we demonstrated, using an in vitro approach, that the pattern recognition Toll-like receptor (TLR)3 plays a key role in the immune response of lung epithelial cells to IAV. In view of these data and the fact that the functional role of TLR3 in vivo is still debated, we designed an investigation to better understand the role of TLR3 in the mechanisms of IAV pathogenesis and host immune response using an experimental murine model. The time-course of several dynamic parameters, including animal survival, respiratory suffering, viral clearance, leukocyte recruitment into the airspaces and secretion of critical inflammatory mediators, was compared in infected wild-type and *TLR3* ^−/−^ mice. First, we found that the pulmonary expression of TLR3 is constitutive and markedly upregulated following influenza infection in control mice. Notably, when compared to wild-type mice, infected *TLR3* ^−/−^ animals displayed significantly reduced inflammatory mediators, including RANTES (regulated upon activation, normal T cell expressed and secreted), interleukin-6, and interleukin-12p40/p70 as well as a lower number of CD8^+^ T lymphocytes in the bronchoalveolar airspace. More important, despite a higher viral production in the lungs, mice deficient in TLR3 had an unexpected survival advantage. Hence, to our knowledge, our findings show for the first time that TLR3-IAV interaction critically contributes to the debilitating effects of a detrimental host inflammatory response.

## Synopsis

Influenza A virus (IAV) is responsible for highly contagious acute respiratory disease. Recent concerns have risen concerning a possible influenza pandemic in the near future. Thus, a better understanding of the molecular mechanisms of IAV pathogenesis and host immune responses is required for the development of more efficient means of prevention and treatment of influenza. The Toll-like receptor (TLR)3 is a member of a family of receptors that detects microbes and triggers host defenses. We previously demonstrated using an in vitro approach, that the TLR3 plays a key role in the response of lung epithelial cells to IAV. Here, we used a mouse model to dissect the in vivo importance of TLR3-dependent responses during influenza. The time-course of several parameters, including animal survival, respiratory distress, viral clearance, and inflammation, was compared in infected control wild-type and TLR3-deficient mice. Our findings reveal that *TLR3* ^−/−^ mice have an unexpected advantage against IAV challenge as we show for the first time that a reduction of TLR3-mediated inflammatory response reduces the clinical manifestations of IAV-induced pneumonia.

## Introduction

Recent outbreaks of highly pathogenic influenza A virus (IAV) infections have had important economic repercussions and have raised concerns that a new influenza pandemic will occur in the near future. The most severe complication of influenza is acute pneumonia, which develops rapidly and may result in respiratory failure and death. The etiological agents of the disease, the single-stranded RNA influenza viruses, are classified into three types (A, B, and C), of which influenza A is clinically the most important [1]. In the United States alone, there are more than 20,000 deaths per year, and in the large pandemic of 1918, over 20 million people died worldwide [2–4]. Although vaccines and antiviral molecules to control influenza have been developed during the last years, the disease is by no means under control since these treatments are not available worldwide and their efficacy is not optimal [3–5]. Thus, a better understanding of the molecular mechanisms of IAV pathogenesis and host immune responses is required for the development of more efficient means of prevention and treatment of influenza.

The invasion of viruses is initially sensed by the host innate immune system, triggering rapid antiviral responses that involve the release of proinflammatory cytokines, and leading to the subsequent activation of adaptive immune responses. Diverse components of infecting viruses can induce the signalling pathways that regulate the cellular antiviral gene program. Among them, double-stranded RNA (dsRNA) has been viewed as the most important component. It is a common signature linked to the viral replication cycle and lysis of virus-infected cells is hypothesized to release dsRNA [6,7]. A major transducer of cell signalling generated by dsRNA is the Toll-like receptor (TLR)3, a member of a conserved family of innate immune recognition receptors that have key roles in detecting microbes, initiating innate immune responses, and linking innate and adaptive immunity [8–10].

A role for TLR3 in viral detection has been suggested by in vitro and ex vivo studies [8,11] but except for a recent study that has clearly shown that TLR3 mediates West Nile virus entry into the brain, causing lethal encephalitis, the functional role of TLR3 in vivo remains unclear [12,13]. Recently, using an in vitro approach, we demonstrated that TLR3 and its signaling-associated molecule TRIF play a key role in the immune response of respiratory epithelial cells to IAV [14]. In the current study, we used TLR3-deficient (*TLR3^−/−^*) mice to study the specific role of this pattern-recognition receptor in IAV-mediated acute pneumonia. Our findings clearly indicate that TLR3 contributes to a detrimental inflammatory response.

## Results

### Kinetics of Pathological Features Associated with IAV Infection

**Figure 1:**
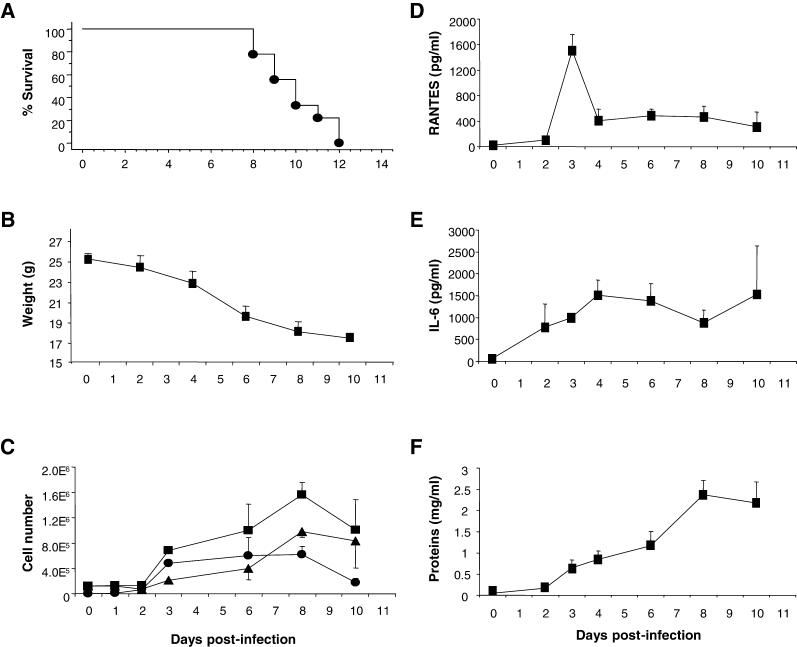
Time-course of Dynamic Parameters in Wild-Type Mice Infected by a Lethal IAV Challenge Male C57Bl/6 mice were infected intranasally with 300 pfu of IAV and different parameters were analyzed during the course of infection. (A) Survival of mice. (B) Body weight changes. (C) Leukocyte recruitment into the airways ▪, all leukocytes; •, polymorphonuclear cells; ▴, mononuclear cells). (D and E) RANTES and IL-6 production in BAL fluids. (F) Total protein amount in BAL fluids as an index of alveolocapillar permeability. All these results are the mean ± SD values obtained from three distinct animals and are representative of three independent experiments.

The pathogenesis and immune response associated with influenza pneumonia are assumed to be very complex [15–17]. To further dissect the role of TLR3 in IAV sensing and pathogenesis, we first characterized in infected wild-type animals the time-course of major dynamic parameters, including animal mortality and weight, leukocyte recruitment into the airspaces, increase in alveolocapillar permeability, and secretion of critical mediators. Figure 1A shows that all C57Bl/6 mice inoculated intranasally with IAV at a dose of 300 plaque-forming units (pfu) per mouse died within 12 d. Signs of piloerection and anorexia were associated with a loss of weight (approximately 32%) and appeared after 4 d of infection (Figure 1B). Animals were killed at different intervals postinfection, and bronchoalveolar lavage (BAL) samples were collected to assess cellular infiltration and mediators content in the airspaces. Figure 1C (square symbol) shows a biphasic leukocyte recruitment constituted mainly of polymorphonuclear cells (circle symbol) by days 3 to 8 postinfection and of mononuclear cells (triangle symbol) afterward. The kinetics of IAV-induced secretion of a major cytokine (IL-6) and a CC chemokine (RANTES) was investigated. While RANTES peaked at day 3 and decreased significantly thereafter (Figure 1D), IL-6 secretion increased steadily until day 4 and was sustained at that level until day 11 (Figure 1E). As an index of transudation from the vascular compartment into the lungs, the amount of total protein was determined in the BAL (Figure 1F). Interestingly, the protein concentration curve parallels that of total leukocyte content (Figure 1C, square symbol) confirming a connection between increased microvascular permeability and cellular infiltration during acute lung injury [18]. BAL fluids were further analyzed by an inflammatory protein array to examine at days 3 and 10 postinfection whether other major components, not measured in the initial assays, were affected during influenza pneumonia. A total of 32 mediators were measured, which included 16 cytokines, 11 chemokines, three growth factors, one metalloproteinase inhibitor, and one soluble cytokine receptor (an example of such protein array blot is shown later in Figure 4A) This array analysis indicated selective major increases of granulocyte colony-stimulating factor (G-CSF), IL-6, monocyte chemoattractant protein-1 (MCP-1), MCP-5, macrophage inflammatory protein-2α, RANTES, soluble tumor necrosis factor-α (TNFα) receptor, and tissue inhibitor of metalloproteinase-1 (not illustrated). Next, we checked that the lethal pneumonia induced by IAV was not associated with a bacteria superinfection, as it has been reported by others [19]. We showed that bacteria counts in blood or BAL of IAV-treated mice at day 3, 6, 8, or 10 postinfection were negligible (constantly below 250 cfu/ml). In addition, intramuscular administration of the mice with a large-spectrum antibiotic (Clamoxyl; amoxicillin trihydrate-potassium clavulanate, 25 mg/kg) before and during the viral infection did not reduce or delay the survival pattern (unpublished data).

**Figure 2:**
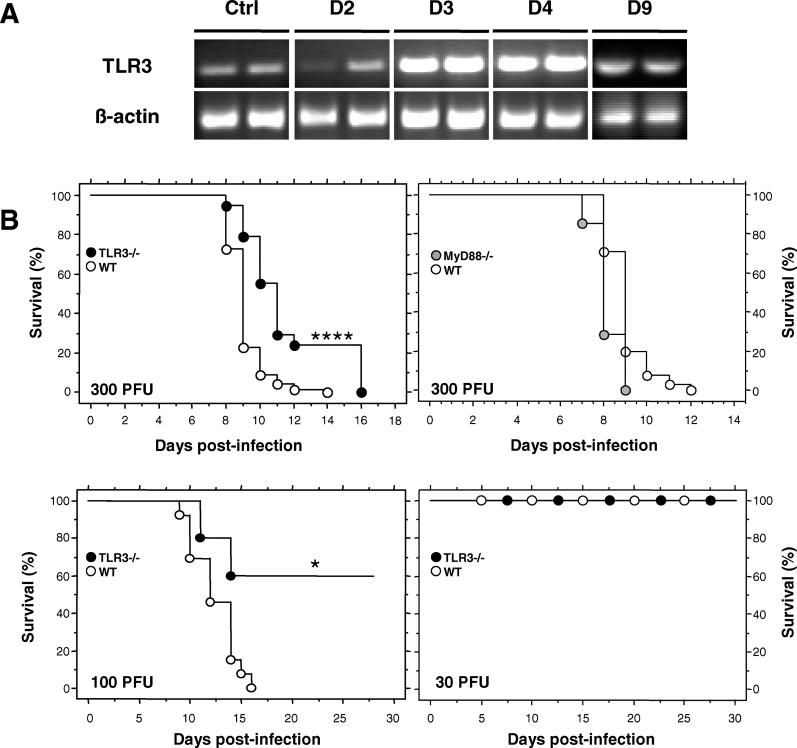
Reduced Lethality in IAV-Infected TLR3^−/−^ Mice (A) Pulmonary expression and regulation of TLR3 during influenza infection. Mice were either noninfected (Ctrl) or infected with 300 pfu of IAV by intranasal route and whole lungs were harvested at 2, 3, 4, and 9 d postinfection. Total RNA was extracted and TLR3 mRNA was analyzed by RT-PCR; a representative result of two is shown. β-Actin mRNA was assessed as a control for RNA loading. (B) Lethality induced by IAV in *TLR3* ^−/−^ and *MyD88* ^−/−^ mice in comparison with wild-type mice. Age-matched *TLR3^−/−^*, *MyD88^−/−^*, and wild-type male mice received intranasally 300, 100, or 30 pfu of IAV. Wilcoxon test for comparisons of Kaplan-Meier survival curves indicated a significant increase in the survival of *TLR3^−/−^* mice compared to that of wild-type animals (****p < 0.0001, *p < 0.05) but not to that of *MyD88^−/−^* mice.

### IAV Upregulates the Lung Expression of TLR3

We previously demonstrated that of all the mediators we tested, i.e., bacterial LPS, the cytokines TNFα and IL-1β, the protein kinase C activator PMA, IAV, and the synthetic dsRNA poly(I:C), only the two latter stimuli upregulated TLR3 expression in human pulmonary epithelial cells, suggesting that the signaling pathways controlling the induction of this gene are restricted [14]. Given the distinct complexity between in vitro and in vivo systems, there was a need to determine the regulation and the function of TLR3 in relation to the pathogenesis of influenza in an experimental animal model. We first showed that the expression of this receptor in the lungs of infected mice is constitutive and markedly upregulated following IAV administration, peaking at day 3 and slightly decreasing at day 9 postinfection (Figure 2A).

### Reduced Inflammation and Lethality in IAV-Infected *TLR3^−/−^* Mice

**Figure 3:**
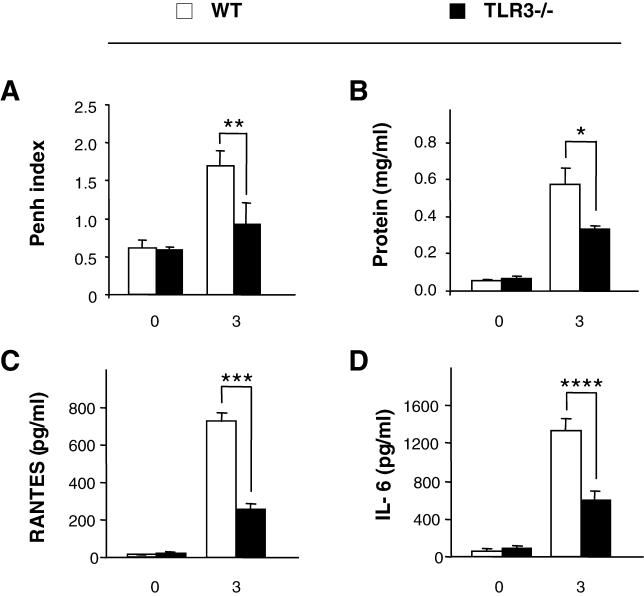
Reduced Inflammation in IAV-Infected *TLR3^−/−^* Mice (A) Basal respiratory function of wild-type versus *TLR3^−/−^* mice before (day 0) and 3 d post–viral infection. This was measured using a barometric plethysmographic chamber and is expressed as Penh (cf. Materials and Methods for details), the increase of which is an indicator of deterioration changes in airway mechanics (**p < 0.01). (B–D) BAL fluids levels of total protein, RANTES, and IL-6 in wild-type versus *TLR3^−/−^* mice before (day 0) and 3 d post–viral infection (*p < 0.05, ***p < 0.001, ****p < 0.0001). Histograms are the mean ± SD values obtained from five animals and are representative of at least three independent experiments.

**Figure 4:**
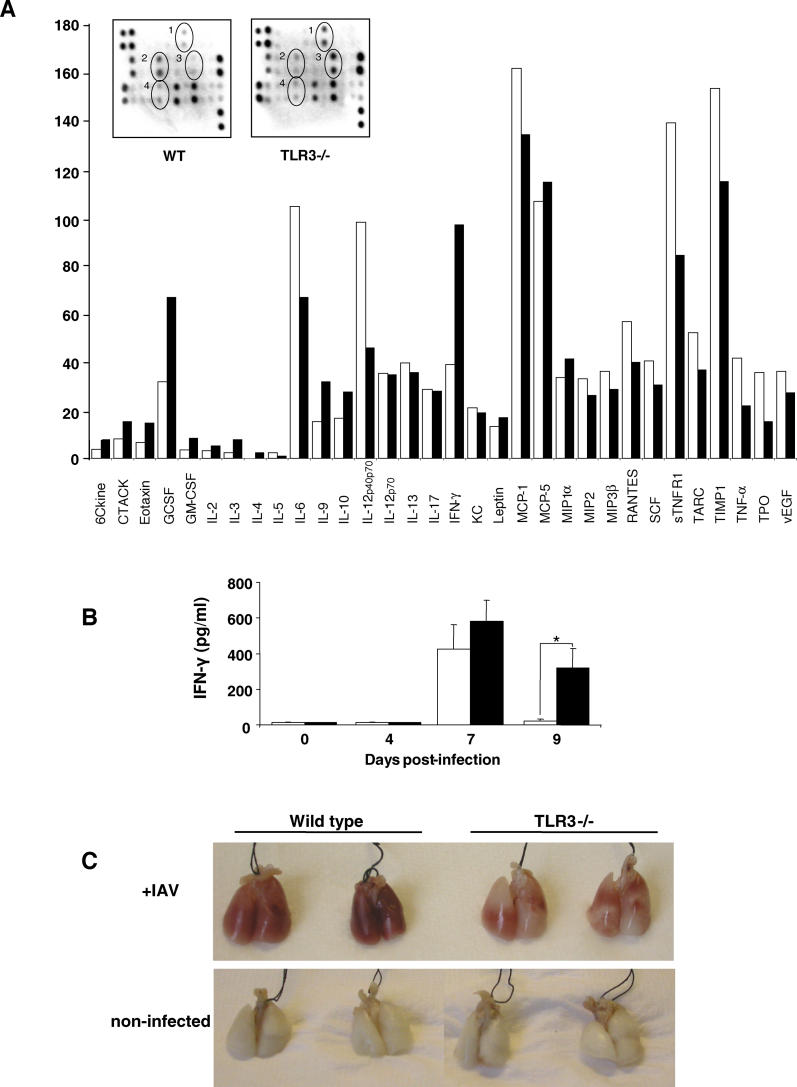
Distinct Inflammatory Profile in Wild-Type and *TLR3^−/−^* Mice Infected by a Lethal IAV Challenge (A) BAL fluids levels of inflammatory mediators at day 9 postinfection. The data were normalized to internal positive controls spotted on the same protein array membrane and are expressed as relative units. (Inset) Example of a protein array blot probed with BAL collected from wild-type and *TLR3^−/−^* mice. Relevant spots are highlighted: (1) G-CSF, (2) IL-12p40/p70, (3) IFN-γ, and (4) RANTES. (B) BAL fluids levels of IFN-γ in wild-type (□) and *TLR3^−/−^* (▪) mice before (day 0) and 4, 7, and 9 d postinfection by 300 pfu IAV (*p < 0.05). Histograms are the mean ± SD values obtained from five animals and are representative of three independent experiments. (C) Examination of lungs isolated from noninfected wild-type and *TLR3^−/−^* mice (lower panel) and at day 9 post–viral infection (upper panel).

**Figure 5:**
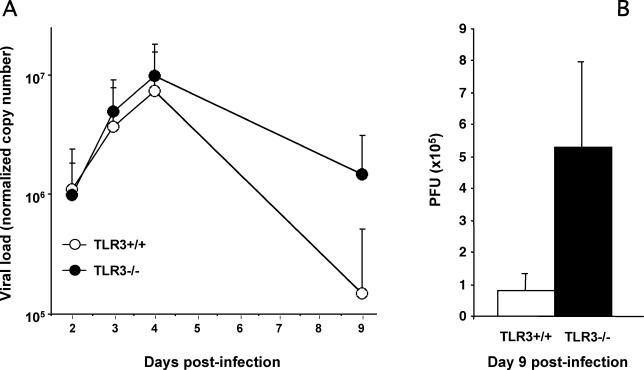
Viral Load in the Lungs of IAV-Infected Mice Viral load in *TLR3^−/−^* and wild-type mice challenged intranasally by 300 pfu of IAV. (A) Results are the mean ± SD values obtained from four animals at days 2 to 4 postinfection and 17 mice at day 9 postinfection. They are expressed as RNA copies normalized to β-actin expression levels, as determined by real-time PCR. (B) Results are the mean ± SD values obtained from nine wild-type and eight *TLR3^−/−^* mice at day 9 postinfection. They are expressed as pfu, as determined by standard plaque assay [21].

Remarkably, we showed that *TLR3^−/−^* mice had a more prolonged survival after a challenge of 300 pfu IAV than did wild-type animals as determined by a Kaplan-Meier test; i.e., approximately 26% and 0% survival, respectively, at day 12 postinfection (*n* = 35 and 65, respectively; *p* < 0.0001, Figure 2B, left upper panel). Ultimately, all *TLR3^−/−^* mice died 3 to 5 d later. We have also performed survival experiments that were conducted with lower doses of virus (100 and 30 pfu; Figure 2B, lower panels). The results clearly show that the absence of TLR3 can undeniably protect the mice from the lethal viral effects. Thus, after a challenge of 100 pfu IAV, *TLR3^−/−^* mice have an even more enhanced survival (approximately 60%; even after a month postinfection; *n* = 5) compared to wild-type animals (0%, *n* = 13). Infection with 30 pfu IAV affected neither the wild-type nor the *TLR3^−/−^* mice (*n* = 5). Interestingly, mice deficient for the adapter protein myeloid differentiation (MyD)88, which is involved in the signaling of all TLR molecules but TLR3, were as sensitive as wild-type mice to influenza infection (0% survival for both groups, Figure 2B, right upper panel; *n* = 8). Then, to monitor the role of TLR3 in lung dysfunction induced by the viral infection, the respiratory distress index Penh and total protein amount as well as the secretion of inflammatory cytokines and chemokines were evaluated in BAL fluids of *TLR3^−/−^* and wild-type animals at day 3 postinfection by 300 pfu IAV (this time point was chosen as it corresponds to the peak of the viral load in the lungs of both animal groups, cf. Figure 5). Figure 3A shows that the Penh index was significantly diminished in *TLR3^−/−^* mice compared to control mice, i.e., 0.95 ± 0.12 versus 1.74 ± 0.2, respectively (*n* = 5, *p* = 0.005). Likewise, total protein, RANTES, and IL-6 amounts were significantly lower in *TLR3^−/−^* than in wild-type mice (Figure 3B–3D). BAL fluids were further analyzed by an inflammatory protein array to examine at day 9 whether the expression of additional mediators was directly regulated by TLR3. Figure 4A not only confirms a clearly reduced IL-6 production in the lungs of *TLR3^−/−^* mice but also reveals a similar inhibition concerning inflammatory mediators such as IL-12p40/p70, sTNFR1, and tissue inhibitor of metalloproteinase-1. On the contrary, the expression of distinct components is increased in *TLR3^−/−^* versus wild-type lungs, including not only INF-γ but also G-CSF and, to a lesser extent, IL-9 and IL-10. As additional evidence, we showed by ELISA that, although similar at day 4 to day 7 post–viral infection, the amount of interferon (IFN)-γ in *TLR3^−/−^* mice was greater (approximately 14.5 times that of wild-type animals) at day 9 (Figure 4B). Finally, Figure 4C presents a typical gross morphological view of perfused lungs isolated from noninfected wild-type and *TLR3^−/−^* mice (lower panel) and at day 9 postinfection by 300 pfu of IAV (upper panel). Lungs of wild-type animals appeared severely injured as manifested by an almost black hemorrhaged lung surface, whereas those obtained from *TLR3^−/−^* mice produced only faintly and diffuse red lungs, suggesting that the lesions induced by IAV are reduced in the absence of TLR3.

### Paradoxical Increased Lung Viral Load in *TLR3^−/−^* Mice

The previous results indicated that a potent inflammatory reaction occurs in the lungs of wild-type mice after influenza infection and that this process is critically reduced or altered in *TLR3* ^−/−^ animals.

Inflammatory signaling pathways during viral infection have been interpreted in some cases as a protective response of the host, whereas in other cases the virus can utilize these pathways to enhance its replication [20]. Thus, we first used quantitative RT-PCR (qRT-PCR) to investigate whether TLR3-dependent host response might regulate the replication of IAV. Figure 5 shows that virus replication was major in both wild-type and *TLR3^−/−^* mice with very similar titers when examined on days 1 to 4 post–viral infection. Paradoxically, while control animals had significantly reduced the virus load of their lungs at day 9 postinfection, the virus persisted in *TLR3* ^−/−^ mice with an elevated amount (approximately nine times that of wild-type animals). Note that we also used a more traditional method to accurately determine IAV potency. Viral replication was studied in MDCK cell lines at day 9 postinfection, according to a standard protocol [21]. We confirmed that *TLR3* ^−/−^ mice had a significantly higher IAV amount in their lungs compared to wild-type animals (*p* < 0.001).

### Wild-Type and *TLR3^−/−^* Mice Raise a Contrasted Leukocyte Content in Their Lungs after Infection by IAV

**Figure 6:**
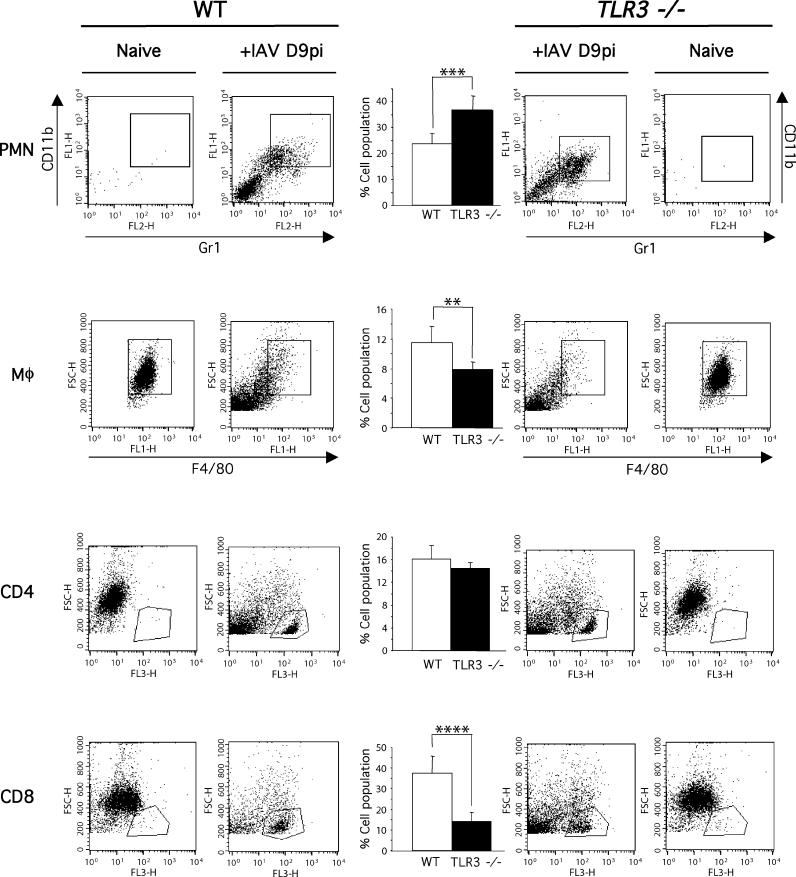
Wild-Type and *TLR3^−/−^* Mice Raise a Contrasted Leukocyte Content in Their Lungs after IAV Challenge BAL cells were collected from IAV-infected mice at day 9 postinfection. To characterize the recovered leukocyte cell types, polymorphonuclear neutrophils (PMN, Gr1^+^, CD11b^+^), macrophages (Mϕ, F4/80^+^), CD4^+^ T lymphocytes (CD4), and CD8^+^ T lymphocytes (CD8) were stained with fluorescently labeled specific antibodies. Far right and far left dot-plots: Representative BAL cell composition of naive wild-type and *TLR3^−/−^* mice. Right and left dot-plots: Representative BAL cell composition of wild-type mice and *TLR3^−/−^* mice at day 9 postinfection by 300 pfu of IAV. Central histograms: Results are the mean ± SD obtained from 12 wild-type mice and seven *TLR3^−/−^* mice (**p < 0.01, ***p < 0.001, ****p < 0.0001).

Some of the foregoing findings suggested that a TLR3-mediated host immune response plays a harmful role in the pathogenesis of IAV infection. In that regard, it is of note that several studies revealed a functional redundancy and synergy of different immune cells in the antiviral response to IAV [15,17,22]. Thus, to gain insight into which cell type can possibly participate in the TLR3-regulated detrimental immune response, leukocytes were harvested from the BAL of wild-type and *TLR3* ^−/−^ mice 9 d postinfection and the number and phenotype of the cells were characterized by flow cytometry. Infection of wild-type mice with IAV resulted in a significant increase in the number of leukocytes (cf. Figure 1C), and the accumulated cells in the BAL were composed mostly of T-lymphocytes (55 × 10^4^ ± 12.5 × 10^4^/BAL), neutrophils (23 × 10^4^ ± 4.1 × 10^4^/BAL), and macrophages (11 × 10^4^ ± 2.2 × 10^4^/BAL) (Figure 6, left and central panels); these numbers have to be compared to a leukocyte population in naïve mice constituted by approximately 90% macrophages (Figure 6, left panels and [23]). The absolute numbers of the whole leukocyte population in the BALs from either infected wild-type or *TLR3* ^−/−^ mice were similar (approximately 1 × 10^6^ cells). Among the T lymphocytes, the CD8^+^ T cells were the predominant cell population in the lungs of infected wild-type animals (37.3 × 10^4^ ± 7.8 × 10^4^/BAL). By contrast, the number of CD8^+^, but not CD4^+^, T lymphocytes in *TLR3^−/−^* mice was considerably lower (about a third of that found in wild-type animals (i.e., 14.5 × 10^4^ ± 4.5 × 10^4^/BAL; *p* = 1 × 10^−6^; Figure 6, right and central panels). Likewise, a significant reduction (approximately 1.5 times) of the number of macrophages was observed in *TLR3^−/−^* mice (i.e., 7.6 × 10^4^ ± 0.99 × 10^4^/BAL; *p* = 0.004), whereas the number of neutrophils was 1.5 times higher in this same group of mice (i.e., 32.9 × 10^4^ ± 4.9 × 10^4^/BAL, *p* = 0.0009; Figure 6, right and central panels).

## Discussion

Our understanding of how the immune system recognizes pathogens has increased exponentially in recent years due to the discovery of the TLR family. TLRs play a central role in the detection of pathogen-associated molecular patterns and in the initiation of an effective innate and adaptive immune response [9,10]. In agreement with this paradigm, there is accumulating in vivo evidence using *TLR^−/−^* mice that support a role for these receptors in antibacterial, antifungal, and antiviral defense. Moreover, several clinical reports confirm the contribution of TLRs to the pathophysiology of infectious diseases, and polymorphisms in TLR genes are associated with predisposition to severe infections [24].

In view of this major information, we anticipated at the start of our investigation that TLR3 would act as a protective component and, conversely, its absence in *TLR3* ^−/−^ mice would render the animals more susceptible to IAV infection. On the contrary, our study reveals that mice deficient in TLR3 have an unexpected advantage. Thus, in comparison with wild-type mice, we found in *TLR3* ^−/−^ animals (i) a clearly reduced level of inflammatory mediators in the bronchoalveolar spaces, including RANTES, IL-6, and IL-12p40/p70; (ii) a lower number of the predominant leukocyte population in the airspaces, i.e., the CD8^+^ T cells; and, most important, (iii) a paradoxical longer survival. Based on these findings and in view of previous work describing the major role of CD8^+^ T lymphocytes and cytokines in the pathogenesis of influenza infection, we discuss below the fact that the enhanced resistance of IAV-infected *TLR3* ^−/−^ might be due to a lower TLR3-mediated release of inflammatory mediators and T cell infiltration in the lung airspaces.

It is established that IAV replicates in epithelial cells and leukocytes, resulting in the production of chemokines and cytokines that favors the recruitment of mononuclear cell population to the site of infection. There is growing evidence that the mediators that have a central role in the resolution of influenza are the same that can be the cause of many clinical signs related to this pathology [25–27]. We confirm in the present study that influenza infection leads to the synthesis of major inflammatory cytokines and chemokines, including IL-6, G-CSF, IL-12p40/p70, MCPs, macrophage inflammatory proteins, and RANTES. Remarkably, IL-6 exhibits multifunctional activities that are largely proinflammatory and its release has been correlated with the symptom pathogenesis during acute influenza [26]. IL-12 administration was also found to have an adverse effect on the course of influenza infection [27]. Other studies reported the involvement of cytokines in influenza pathogenesis, but the blockade of one individual cytokine appears somewhat partial as there is a substantial redundancy between cytokines [15,17]. Notably, our study clearly establishes that TLR3 plays a major role in the inflammatory cytokine response to IAV, the lack of TLR3 resulting in a significant decrease of cytokine synthesis, including that of IL-6, IL-12p40/p70, and RANTES. Ongoing loss-of-function in vitro studies with human pulmonary epithelial cells also demonstrate an essential function for TLR3 in the production of inflammatory cytokines, including IL-6, after IAV challenge (unpublished data). On the contrary, the expression of some other mediators is increased in *TLR3* ^−/−^ versus wild-type lungs. The consequences and the mechanisms of this TLR3-regulated differential expression of cytokines and chemokines are rather complex to hypothesize at this stage due to the pleiotropic and multiple effects of these mediators on diverse cell types. Moreover, these molecules induce or inhibit the production of other cytokines or mediators from their targets in a complex array of positive and negative feedback loops [15,17].

Regardless, consistent with the role of TLR3 in virus-induced inflammation, two of us recently established that infection of *TLR3* ^−/−^ mice with the West Nile virus induces a lower secretion of cytokines, including IL-6 and TNF-α, compared to wild-type mice, that leads to reduced neuronal injury and increased survival in *TLR3* ^−/−^ mice [13]. Similarly, in a study examining the role of another TLR in the host response to herpes simplex virus, Kurt-Jones et al. [28] demonstrated an attenuated cytokine response parallel to a reduction in symptoms of encephalitis in *TLR2* ^−/−^ compared to control animals. It is of note, however, that our results still demonstrate high mortality when the *TLR3* ^−/−^ strain was infected by a high viral amount of IAV, although the difference between this group and wild-type animals is highly significant (*p* < 0.0001). This finding suggests that TLR3 contributes to the shaping of a harmful innate and adaptive immune response at an extent that varies under the IAV load, i.e., that supplementary signaling pathways may be involved at high viral load. In that regard, it is of note that while TLR3 has emerged as a key sensor of viral dsRNA, very recent studies show that cells also express RNA helicases that function as alternative pattern-recognition receptors that detect actively replicating viruses in cytoplasm (reviewed in [29]). Hence, depending on the IAV dose, it is tempting to speculate that the activity of one dsRNA receptor or the other may be transiently switched on or off. The in vivo evidence that RNA helicases–IAV interaction contributes to the debilitating effects of a detrimental inflammation awaits further studies.

Interestingly, cytokines play critical roles in shaping subsequent adaptive T cell responses. This is the case for IFN-γ, which is generally considered as essential for the control of many microbial infections, and its important role in the antiviral immune defense is highlighted by the fact that several viruses encode proteins designed to interfere with IFN-γ signaling [30]. However, most current evidence studies suggest that it may also exert a strong negative effect on the CD8^+^ T cell, a critical cellular component of the adaptive immune response. Thus, T cells treated with IFN-γ show reduced proliferation and/or increased apoptosis [31]. Moreover, IFN-γ can act as a negative feedback regulator to control Th1-mediated immune responses [32]. In view of those data, it may be conceivable that the excessive IFN-γ recovered from the airspaces of *TLR3* ^−/−^ mice might contribute to a decrease in CD8^+^ T cell number and to a limitation of T cell–mediated inflammation in this group of animals, in comparison with wild-type mice. Indeed, although the recruitment of CD8^+^ T cells is essential for protective responses, it is becoming increasingly evident that they can also be associated with the development of influenza-related immunopathological sequelae [33,34]. We confirmed that CD8^+^ T cells are prominent in the airways of IAV-challenged wild-type mice. The drastic decrease in CD8^+^ T lymphocyte infiltration concomitant with the prolonged survival of the *TLR3^−/−^* mice suggests that a dysregulated TLR3-dependent CD8^+^ T cell response may lead to sustained lung injury. Accordingly, this idea sheds new light on previous studies that have reported that mice given antilymphocyte serum [35] or the immunosuppressive drug cyclophosphamide had a lesser pulmonary pathology when infected with IAV than untreated mice [36,37]. Moreover, numerous studies reported that athymic nude mice exhibited an increased survival time compared to immunocompetent mice given influenza challenge [38–40]. Other authors have more specifically suggested that CD8^+^ T lymphocytes exacerbate the influenza pathology and cause mortality at high viral infection [41]. Clinical observations also lend support to the fact that CD8^+^ T lymphocytes may contribute to the pathological manifestations of IAV infection [42]. Of note is that the detrimental role of the cell-mediated immune response during influenza is analogous to infection with the prototypic arenavirus lymphocytic choriomeningitis virus. Indeed, immunodepressed mice infected with this virus do not die as do immunocompetent mice, despite persistent viral production [43]. This latter feature is especially meaningful in regard to the inability of *TLR3^−/−^* mice as well as athymic animals [38–40] to clear the IAV, whereas they live longer than control animals.

Overall, it emerges that the etiology and pathogenesis of a very complex disease such as influenza likely involve a combination of both virus effects per se and the imbalance between the beneficial and harmful effects of mediators released by immune cells. Moreover, a clear conclusion from our work is that a reduction in TLR3-mediated inflammatory infiltrate reduces the clinical manifestations of IAV-induced pneumonia. Nonetheless, although potentially valuable, there would be no overall benefit from treating influenza solely with TLR3 downregulators as we found that the lack of TLR3 resulted in increased pulmonary IAV load. Hence, while vaccination should remain the basis for influenza prophylaxis [44], we suggest that along with inhibitors of viral replication, a concomitant treatment with modulators of TLR3 might be useful in the management of IAV infections, particularly in at-risk groups during periods when there is a mismatch between the epidemic strain and the vaccine strain.

## Materials and Methods

### Virus preparation and quantification.

Influenza A/Scotland/20/74 (H3N2) virus was generously provided by N. Escriou and S. Van Der Werf (Unité de Génétique Moléculaire des Virus Respiratoires, Institut Pasteur, Paris, France). The virus was prepared as previously described [14]. Viral RNA was isolated from the viral stock with an RNeasy mini-kit (Qiagen, Hilden, Germany) and quantified with a Nanodrop ND-1000 spectrophotometer (Rockland, Delaware, United States). cDNA derived from the viral RNA sample was used as a standard for a qRT-PCR. qRT-PCR was then performed using specific primers (sense: 5′ AAG ACC AAT CCT GTC ACC TCT GA 3′ and antisense: 5′ CAA AGC GTC TAC GCT GCA GTC C 3′; Proligo, Evry, France) that complement 20 temporally and spatially divergent influenza A matrix protein gene sequences, as previously described [45].

### Mouse strains.

Males C57Bl/6 mice were purchased from the Centre d'Elevage R. Janvier (Le Genest Saint-Isle, France) and were used at about 8 wk of age. *MyD88^−/−^* and *TLR3^−/−^* mice were generated as described earlier [8,46]. *MyD88^−/−^* mice were obtained from Dr. S. Akira (Osaka University, Osaka, Japan). Each type of mice was backcrossed at least eight times with C57Bl/6 to ensure similar genetic backgrounds. Mice strains were bred in an animal facility in pathogen-free conditions. Mice were fed normal mouse chow and water ad libitum and were reared and housed under standard conditions with air filtration. For experiments of infection by IAV, mice were housed in cages inside stainless steel isolation cabinets that were ventilated under negative pressure with HEPA-filtered air. Mice were treated in accordance with Pasteur Institute guidelines in compliance with the European animal welfare regulation.

### Animal fluid collection.

Mice were anesthetized by a mixture of ketamine-xylazine (1 and 0.2 mg per mouse, respectively) and infected intranasally with 50 μl of PBS containing 300, 100, or 30 pfu IAV. Mice were observed daily for signs of morbidity. Alternatively, mice were killed at different time points by intraperitoneal injection of 300 mg/kg sodium pentobarbital, and 1 ml of heparinized blood was collected by the vena cava. After centrifugation at 300*g*, the resulting plasma was stored. Airways were washed twice with 1 ml of saline, and the BAL was collected to further determine cell differential counts and percentages using a Coulter counter (Coulter-Electronics, Margency, France) as well as a Diff-Quik staining (Baxter-Dale, Dudingen, Germany) of cytospin slides. Aliquots of BAL fluids were stored for total protein and cytokine measurement. For flow cytometry analysis, cells were eventually counted and stabilized with Cyto-Chex (Sireck, Omaha, Nebraska, United States). Under these conditions, cells could be stored for 1 wk at 4 °C before labeling for flow cytometry analysis.

### Monoclonal antibodies for flow cytometry analysis.

MAbs reactive to CD11b (Mac-1, M1/70, rat IgG2a), Ly-6G-Gr1 (clone RB6-8C5, rat IgG2b), CD8 (clone 53–6.7, rat IgG2a), and CD4 (clone RM4–5, rat IgG2a) were purchased from BD PharMingen (San Diego, California, United States) as conjugated to fluorescein isothiocyanate, phycoerythrin, or cy-Chrome. Phycoerythrin-conjugated F4/80 (clone C1:A3–4, rat IgG-2b) was purchased from Caltag Laboratories, Burlingame, California, United States. Before flow cytometry analysis, cells were washed in PBS containing 5% FCS and stained for 30 min at 4 °C with the conjugated Abs. Cells were further washed twice and analyzed on a FACScan flow cytometer (BD PharMingen).

### RANTES, IL-6, and IFN-γ ELISA.

Murine RANTES, IL-6, and IFN-γ concentrations in BAL were determined using DuoSet ELISA kits obtained from R&D Systems (Minneapolis, Minnesota, United States).

### Inflammatory protein array.

A commercial antibody-based protein array designed to detect 32 inflammatory mediators was used according to the manufacturer's instructions (RayBio Mouse Cytokine Array II; RayBiotech, Atlanta, Georgia, United States). Membrane arrays were hybridised with BAL fluids to compare different types of mice and different time points and were always processed simultaneously. Array images were recorded after amplification with an Ultra-Lum system (Ultra-Lum, Claremont, California, United States), and all scanned images accurately reproduced spots seen on films.

### RT-PCR.

RNA was extracted from the lung tissue using the FastRNA GREEN Kit and FastPrep instrument (Bio 101, Qbiogene, California, United States) according to the protocol from the manufacturer. RNA was reverse transcribed using 0.5 μg of total RNA. PCR was performed using specific primers (Proligo, Evry, France) for mouse TLR3 (sense: 5′ GCT CAT TCT CCC TTG CTC AC 3′; antisense: 5′ CCC GAA AAC ATC CTT CTC AA 3′). As an internal control, we used primers for mouse β-actin (sense: 5′ GGA CTC CTA TGT GGG TGA CGA GG 3′; antisense 5′ GGG AGA GCA TAG CCC TCG TAG AT 3′). Amplifications were performed in a Peltier thermal cycler apparatus (MJ Research, Watertown, Massachusetts, United States) using the Qbiotaq polymerase (Qbiogene, Illkirch, France). To detect mTLR3, the thermocycling protocol was 95 °C for 3 min, 35 cycles of denaturation at 95 °C for 30 s, annealing at 58 °C for 30 s, and extension at 72 °C for 1 min. To detect β-actin, only 30 cycles were used and the annealing temperature was 62 °C. Amplification products were resolved on 1.5% agarose gel containing ethidium bromide. Band intensities on gels were recorded after amplification with an Ultra-Lum system. Samples for each point were serially diluted to verify that PCR was performed in the linear phase of the amplification reaction (unpublished data).

### Assessment of the basal respiratory function.

Unrestrained conscious mice, infected or not by IAV, were placed in a whole body plethysmographic chamber (Buxco Electronics, Sharon, Connecticut, United States) to analyze the respiratory waveforms. The system measures both the magnitude and the slope of the chamber pressure. After a 10-min stabilization period, the basal respiratory capacity of each individual mouse was estimated by recording the enhanced pause pressure expressed as “Penh,” calculated as Penh = [*Te* (expiratory time)/*Tr* (relaxation time)] − 1 × [*Pef* (peak expiratory flow)/*Pif* (peak inspiratory flow)]. The values of Penh expressed per minute were averaged from three determinations recorded every 20 s. A Penh increase is an indicator of deterioration in airway mechanics.

### Statistical analysis.

Statistical significance between the individual groups was analyzed using the unpaired Student's *t* test with a threshold of *p* < 0.05. Survival of mice was compared using Kaplan-Meier analysis and log-rank test.

## Supporting Information

### Accession Numbers

The Entrez (http://www.ncbi.nlm.nih.gov/Entrez) accession number for the TLR3 gene is BC099937, and the UniProt (http://www.uniprot.org) accession number for the TLR3 protein is Q99MB1.

## Abbreviations

BAL: bronchoalveolar lavage
dsRNA: double-stranded RNA
G-CSF: granulocyte colony-stimulating factor
IAV: influenza A virus
IFN: interferon
IL: interleukin
MCP: monocyte chemoattractant protein
MyD: myeloid differentiation
pfu: plaque-forming units
qRT-PCR: quantitative RT-PCR
RANTES: regulated upon activation, normal T cell expressed and secreted
TLR: Toll-like receptor
TNF: tumor necrosis factor

## References

[ppat-0020053-b001] Wright PF, Webster R, Knipe DM, Howley PM (2001). Orthomyxoviruses. Fields virology, 4th edition.

[ppat-0020053-b002] Cheung CY, Poon LL, Lau AS, Luk W, Lau YL (2002). Induction of proinflammatory cytokines in human macrophages by influenza A (H5N1) viruses: A mechanism for the unusual severity of human disease?. Lancet.

[ppat-0020053-b003] Kandel R, Hartshorn KL (2005). Novel strategies for prevention and treatment of influenza. Expert Opin Ther Targets.

[ppat-0020053-b004] Palese P (2004). Influenza: Old and new threats. Nat Med.

[ppat-0020053-b005] Hayden FG (2004). Pandemic influenza: Is an antiviral response realistic?. Pediatr Infect Dis J.

[ppat-0020053-b006] Karpala AJ, Doran TJ, Bean AG (2005). Immune responses to dsRNA: Implications for gene silencing technologies. Immunol Cell Biol.

[ppat-0020053-b007] Sen GC, Sarkar SN (2005). Transcriptional signaling by double-stranded RNA: Role of TLR3. Cytokine Growth Factor Rev.

[ppat-0020053-b008] Alexopoulou L, Holt AC, Medzhitov R, Flavell RA (2001). Recognition of double-stranded RNA and activation of NF-kappaB by Toll-like receptor 3. Nature.

[ppat-0020053-b009] Finberg RW, Kurt-Jones EA (2004). Viruses and Toll-like receptors. Microbes Infect.

[ppat-0020053-b010] Bowie AG, Haga IR (2005). The role of Toll-like receptors in the host response to viruses. Mol Immunol.

[ppat-0020053-b011] Tabeta K, Georgel P, Janssen E, Du X, Hoebe K (2004). Toll-like receptors 9 and 3 as essential components of innate immune defense against mouse cytomegalovirus infection. Proc Natl Acad Sci U S A.

[ppat-0020053-b012] Edelmann KH, Richardson-Burns S, Alexopoulou L, Tyler KL, Flavell RA (2004). Does Toll-like receptor 3 play a biological role in virus infections?. Virology.

[ppat-0020053-b013] Wang T, Town T, Alexopoulou L, Anderson JF, Fikrig E (2004). Toll-like receptor 3 mediates West Nile virus entry into the brain causing lethal encephalitis. Nat Med.

[ppat-0020053-b014] Guillot L, Le Goffic R, Bloch S, Escriou N, Akira S (2005). Involvement of Toll-like receptor 3 in the immune response of lung epithelial cells to double-stranded RNA and influenza A virus. J Biol Chem.

[ppat-0020053-b015] Julkunen I, Sareneva T, Pirhonen J, Ronni T, Melen K (2001). Molecular pathogenesis of influenza A virus infection and virus-induced regulation of cytokine gene expression. Cytokine Growth Factor Rev.

[ppat-0020053-b016] Tamura S, Kurata T (2004). Defense mechanisms against influenza virus infection in the respiratory tract mucosa. Jpn J Infect Dis.

[ppat-0020053-b017] Van Reeth K (2000). Cytokines in the pathogenesis of influenza. Vet Microbiol.

[ppat-0020053-b018] Chignard M, Balloy V (2000). Neutrophil recruitment and increased permeability during acute lung injury induced by lipopolysaccharide. Am J Physiol Lung Cell Mol Physiol.

[ppat-0020053-b019] Hussell T, Williams A (2004). Menage a trois of bacterial and viral pulmonary pathogens delivers coup de grace to the lung. Clin Exp Immunol.

[ppat-0020053-b020] Santoro MG, Rossi A, Amici C (2003). NF-kappaB and virus infection: Who controls whom?. EMBO J.

[ppat-0020053-b021] Youil R, Su Q, Toner TJ, Szymkowiak C, Kwan WS (2004). Comparative study of influenza virus replication in Vero and MDCK cell lines. J Virol Methods.

[ppat-0020053-b022] Bot A, Reichlin A, Isobe H, Bot S, Schulman J (1996). Cellular mechanisms involved in protection and recovery from influenza virus infection in immunodeficient mice. J Virol.

[ppat-0020053-b023] Lagranderie M, Nahori MA, Balazuc AM, Kiefer-Biasizzo H, Lapa e Silva JR (2003). Dendritic cells recruited to the lung shortly after intranasal delivery of Mycobacterium bovis BCG drive the primary immune response towards a type 1 cytokine production. Immunology.

[ppat-0020053-b024] Schroder NW, Schumann RR (2005). Single nucleotide polymorphisms of Toll-like receptors and susceptibility to infectious disease. Lancet Infect Dis.

[ppat-0020053-b025] Schmitz N, Kurrer M, Bachmann MF, Kopf M (2005). Interleukin-1 is responsible for acute lung immunopathology but increases survival of respiratory influenza virus infection. J Virol.

[ppat-0020053-b026] Kaiser L, Fritz RS, Straus SE, Gubareva L, Hayden FG (2001). Symptom pathogenesis during acute influenza: Interleukin-6 and other cytokine responses. J Med Virol.

[ppat-0020053-b027] Kostense S, Sun WH, Cottey R, Taylor SF, Harmeling S (1998). Interleukin 12 administration enhances Th1 activity but delays recovery from influenza A virus infection in mice. Antiviral Res.

[ppat-0020053-b028] Kurt-Jones EA, Chan M, Zhou S, Wang J, Reed G (2004). Herpes simplex virus 1 interaction with Toll-like receptor 2 contributes to lethal encephalitis. Proc Natl Acad Sci U S A.

[ppat-0020053-b029] Kawai T, Akira S (2006). Innate immune recognition of viral infection. Nat Immunol.

[ppat-0020053-b030] Alcami A, Smith GL (1996). Receptors for gamma-interferon encoded by poxviruses: implications for the unknown origin of vaccinia virus. Trends Microbiol.

[ppat-0020053-b031] Refaeli Y, Van Parijs L, Alexander SI, Abbas AK (2002). Interferon gamma is required for activation-induced death of T lymphocytes. J Exp Med.

[ppat-0020053-b032] Feuerer M, Eulenburg K, Loddenkemper C, Hamann A, Huehn J (2006). Self-limitation of Th1-mediated inflammation by IFN-γ. J Immunol.

[ppat-0020053-b033] Liu F, Whitton JL (2005). Cutting edge: Re-evaluating the in vivo cytokine responses of CD8^+^ T cells during primary and secondary viral infections. J Immunol.

[ppat-0020053-b034] Wiley JA, Cerwenka A, Harkema JR, Dutton RW, Harmsen AG (2001). Production of interferon-gamma by influenza hemagglutinin-specific CD8 effector T cells influences the development of pulmonary immunopathology. Am J Pathol.

[ppat-0020053-b035] Suzuki F, Oya J, Ishida N (1974). Effect of antilymphocyte serum on influenza virus infection in mice. Proc Soc Exp Biol Med.

[ppat-0020053-b036] Hurd J, Heath RB (1975). Effect of cyclophosphamide on infections in mice caused by virulent and avirulent strains of influenza virus. Infect Immun.

[ppat-0020053-b037] Singer SH, Noguchi P, Kirschstein RL (1972). Respiratory diseases in cyclophosphamide-treated mice. II. Decreased virulence of PR8 influenza virus. Infect Immun.

[ppat-0020053-b038] Sullivan JL, Mayner RE, Barry DW, Ennis FA (1976). Influenza virus infection in nude mice. J Infect Dis.

[ppat-0020053-b039] Wyde PR, Couch RB, Mackler BF, Cate TR, Levy BM (1977). Effects of low- and high-passage influenza virus infection in normal and nude mice. Infect Immun.

[ppat-0020053-b040] Wells MA, Albrecht P, Ennis FA (1981). Recovery from a viral respiratory infection. I. Influenza pneumonia in normal and T-deficient mice. J Immunol.

[ppat-0020053-b041] Moskophidis D, Kioussis D (1998). Contribution of virus-specific CD8^+^ cytotoxic T cells to virus clearance or pathologic manifestations of influenza virus infection in a T cell receptor transgenic mouse model. J Exp Med.

[ppat-0020053-b042] Enelow RI, Mohammed AZ, Stoler MH, Liu AN, Young JS (1998). Structural and functional consequences of alveolar cell recognition by CD8^+^ T lymphocytes in experimental lung disease. J Clin Invest.

[ppat-0020053-b043] McGavern DB, Homann D, Oldstone MB (2002). T cells in the central nervous system: the delicate balance between viral clearance and disease. J Infect Dis.

[ppat-0020053-b044] Uhnoo I, Linde A, Pauksens K, Lindberg A, Eriksson M (2003). Treatment and prevention of influenza: Swedish recommendations. Scand J Infect Dis.

[ppat-0020053-b045] Ward CL, Dempsey MH, Ring CJ, Kempson RE, Zhang L (2004). Design and performance testing of quantitative real time PCR assays for influenza A and B viral load measurement. J Clin Virol.

[ppat-0020053-b046] Adachi O, Kawai T, Takeda K, Matsumoto M, Tsutsui H (1998). Targeted disruption of the MyD88 gene results in loss of IL-1- and IL-18-mediated function. Immunity.

